# Conservation of Nef function across highly diverse lineages of SIVsmm

**DOI:** 10.1186/1742-4690-6-36

**Published:** 2009-04-09

**Authors:** Jan Schmökel, Hui Li, Elizabeth Bailes, Michael Schindler, Guido Silvestri, Beatrice H Hahn, Cristian Apetrei, Frank Kirchhoff

**Affiliations:** 1 Institute of Virology, University of Ulm, 89081 Ulm, Germany; 2 Departments of Medicine and Microbiology, University of Alabama at Birmingham, Birmingham, Alabama 35294, USA; 3 Institute of Genetics, University of Nottingham, Queens Medical Centre NH7 2UH, Nottingham, UK; 4 Yerkes Regional Primate Research Center, Emory University, Atlanta, Georgia, USA; 5 Department of Pathology and Laboratory Medicine, University of Pennsylvania, Philadelphia, Pennsylvania 19107, USA; 6 Division of Microbiology, Tulane National Primate Research Center, Covington, LA 70433, USA; 7 Heinrich-Pette-Institut, 20251 Hamburg, Germany

## Abstract

**Background:**

SIVsmm is a simian immunodeficiency virus that persists efficiently without causing disease in naturally infected sooty mangabeys (SMs) but induces AIDS upon cross-species transmission to humans and macaques. Current phylogenetic data indicate that SIVsmm strains comprise a highly diverse group of viruses that can be subdivided into different lineages. Since only certain SIVsmm strains have successfully crossed the species barrier to humans and macaques, the question has been raised whether there are lineage specific differences in SIVsmm biology. In the present study we examined whether representatives of five different SIVsmm lineages show differences in the function of the accessory Nef protein, which plays an important role in viral persistence, transmission and pathogenesis.

**Results:**

We found that *nef *alleles from all SIVsmm lineages down-modulated CD4, MHC-I, CD28 and CD3 and up-regulated the invariant chain (Ii) associated with immature MHC-II molecules in human-derived cells. Moreover, they generally suppressed the responsiveness of virally infected T cells to activation, enhanced virion infectivity and promoted virus replication in human peripheral blood mononuclear cells. The functional activity of these *nef *alleles in the various assays varied substantially between different strains of SIVsmm but quantitative analyses did not reveal any significant lineage-specific differences in Nef function.

**Conclusion:**

*Nef *alleles from different lineages of SIVsmm do not require adaptive changes to be functionally active in human cells. Strain rather than lineage-specific differences in Nef function may impact the virological and immunological feature of SIVsmm in SMs and possibly affected viral fitness and pathogenicity in human and macaque hosts.

## Background

To date primate lentiviruses have been detected in about 40 African non-human primate species [1,2]. Two of these viruses, SIVcpz from chimpanzees (*Pan troglodytes troglodytes*) and SIVsmm from sooty mangabeys (SMs) (*Cercocebus atys*) have been transmitted to humans and generated the human immunodeficiency viruses (HIV) types 1 and 2, respectively [3,4]. SIVcpz strains are known to have crossed the species barrier on three occasions, generating HIV-1 groups M, N and O. In contrast, SIVsmm has been transmitted to humans no fewer than eight times [5,6]. Nonetheless, HIV-2 is much less prevalent than HIV-1, with only two transmissions (leading to HIV-2 groups A and B) resulting in significant secondary spread in the human population [7-9]. The remaining transmissions appear to have caused dead-end infections affecting only a handful of individuals [9-11]. SIVsmm was also inadvertently transmitted to captive macaques, generating SIVmac. Currently, experimental infection of macaques with SIVmac is commonly used as a model for studies of AIDS pathogenesis and vaccines [12].

SIVsmm exhibits a prevalence of about 60% in the wild [6] and comprises a genetically highly diverse group of viruses [13]. Previous studies suggest that different SIVsmm strains may differ in their fitness and pathogenic features after cross-species transmission. As mentioned above, only groups A and B of HIV-2 resulted in epidemics. Furthermore, SIVmac strains differ substantially in their ability to persist efficiently and to cause disease in infected rhesus macaques [14,15]. It has been shown that serial passage of SIVsmm in macaques increases viral pathogenicity in this experimental host [16,17]. Thus, differences in viral adaptation to human or macaque hosts may play a role in the ability of this virus to persist and cause disease after cross-species transmission [18-20]. However, intrinsic differences in viral properties may also exist. For example, it has recently been suggested that different SIVsmm lineages vary in their ability to cause a significant loss of CD4^+ ^T cells [21] that is observed in about 10 to 15% of the naturally infected SMs [22]. Lineage-specific differences in viral fitness may also have contributed to the differential spread of the various groups of HIV-2 in the human population.

One viral factor that plays an important role in the efficiency of primate lentiviral persistence and transmission is the Nef protein. Nef performs multiple activities, such as modulation of cell surface expression of CD4, CD28, class I MHC (MHC-II) and the invariant chain (Ii) associated with immature MHC-II molecules, as well as enhancement of viral infectivity and replication [23-29]. In addition, most SIV and HIV-2 Nefs also down-modulate CD3, a key component of the T cell receptor (TCR) complex from the cell surface [30]. It is well established that differences in Nef function affect the virological, immunological and clinical outcome of HIV and SIV infection [31]. Perhaps most importantly, the lack of a functional *nef *gene is associated with very low viral loads and an attenuated clinical course in HIV-1-infected humans [32-34] and SIVmac-infected rhesus macaques [35]. Some HIV-1 and SIVmac strains that contain naturally occurring point mutations or small deletions in Nef are less virulent [36-39], while other alterations in Nef are associated with acutely fatal disease in SIV-infected macaques [40,41]. Recently, it has been shown that inefficient Nef-mediated down-modulation of CD3 and MHC-I correlates with low CD4^+ ^T cell counts in SIVsmm-infected SMs [42].

It has been shown that primary SIVsmm *nef *alleles are functionally active in human-derived cells [42,43]. It is currently unknown, however, whether SIVsmm shows lineage-specific differences in Nef function that may affect virus replication or pathogenicity. To address this question we performed a comprehensive functional analysis of *nef *alleles derived from five different lineages of SIVsmm. We found that all *nef *alleles were capable of modulating cell surface expression of human CD4, CD28, CD3, MHC-I and Ii molecules. Furthermore, they enhanced virion infectivity, promoted viral replication and suppressed the responsiveness of virally infected T cells to activation. Although the magnitude of these various Nef functions varied, we did not find significant lineage-specific differences.

## Methods

### Animals

Blood samples were collected from seven naturally infected SMs housed at the Tulane National Primate Research Center (TNPRC), which represented five different SIVsmm lineages (Ls) based on previous analyses: L1 (M919, M923), L2 (M926, M946), L3 (M949, M951) and L4 (G932) (summarized in Table 1). One L5 SIVsmm strain was isolated on SM PBMC from an animal (FTq) housed at the Yerkes National Primate Research Center (YNPRC) of Emory University. We also included data derived from 22 naturally SIVsmm-infected SMs with differential CD4^+ ^T cell counts housed at the YNPRC [42]. All SMs were maintained in accordance with NIH guidelines. The identification and characterization of the different lineages of SIVsmm has been described [13,21].

### Nef alleles and proviral constructs

SIVsmm *nef *alleles were amplified by RT-PCR from the plasma of seven naturally infected SMs or the supernatant of an SIVsmm FTq infected SM PBMC culture as described previously [42]. Splice-overlap-extension PCR was used to replace the NL4-3 *nef *gene of HIV-1 (NL4-3 based) proviral constructs carrying functional *nef *genes followed by an internal ribosome entry site (IRES) [30] with the bulks of SIVsmm *nef *genes [42]. Cloning and transformation efficiencies were determined and the integrity of all PCR-derived inserts was confirmed by sequence analysis as reported previously [42]. For comparison, we also sequenced the *nef *coding region of three individual clones from each of the proviral plasmids expressing bulk SIVsmm *nef *alleles. The control HIV-1 NL4-3-IRES-eGFP constructs expressing the NL4-3, NA7 and SIVmac239 Nefs or containing a disrupted *nef *gene (*nef*-) and the amplification and functional analysis of *nef *alleles from 22 naturally SIVsmm-infected SMs housed at the YNPRC have been reported previously [42].

### Cell culture and virus stocks

Jurkat and 293T cells were cultured as described previously [30]. Briefly, 293T cells were maintained in Dulbecco's modified Eagle's medium containing 10% heat-inactivated fetal bovine serum. PBMC from healthy human donors were isolated using lymphocyte separation medium (Biocoll Separating Solution, Biochrom), stimulated for 3 days with PHA (1 μg/ml) and cultured in RPMI1640 medium with 10% FCS and 10 ng/ml IL-2 prior to infection. To generate viral stocks, 293T cells were transfected either with the proviral HIV-1 constructs alone (to measure viral infectivity or replication) or cotransfected with a plasmid (pHIT-G) expressing the Vesicular Stomatitis Virus G protein (VSG-G) for flow cytometric analyses [44]. The medium was changed after overnight incubation and the virus was harvested 24 h later. Residual cells in the supernatants were pelleted and the supernatants were stored at -70°C. Virus stocks were quantified using a p24 antigen capture assay provided by the NIH AIDS Research and Reference Reagent Program.

### Transduction and flow cytometry

Jurkat T cells or PBMC were transduced with HIV-1 (NL4-3) constructs coexpressing eGFP and various *nef *alleles and CD4, TCR-CD3, MHC-I, CD28, CD25, CD69 and eGFP expression was measured as described [30,42]. For quantification of Nef-mediated modulation of specific surface molecules, the levels of receptor expression (red fluorescence) were determined for cells expressing a specific range of eGFP. The extent of down-modulation (n-fold) was calculated by dividing the MFI obtained for cells infected with the *nef*-minus NL4-3 control viruses by the corresponding values obtained for cells infected with viruses coexpressing Nef and eGFP.

### NFAT assay

Jurkat cells stably transfected with an NFAT-dependent reporter gene vector [45] were either left uninfected or transduced with HIV-1 Nef/eGFP constructs expressing various *nef *alleles. Except for those cells used as controls, cultures were treated with PHA (1 μg/ml; Murex). Luciferase activity was measured and n-fold induction determined by calculating the ratio between measured relative light units of treated samples over untreated samples as described previously [30].

### Viral infectivity

Virus infectivity was determined using P4-CCR5, TZM-bl and CEM-M7 cells as described [46]. Briefly, the cells were sown out in 96-well-dishes in a volume of 100 μ1 and infected after overnight incubation with virus stocks containing 1 ng of p24 antigen produced by transiently transfected 293T cells. Two days post-infection viral infectivity was detected using the Gal screen kit from TROPIX as recommended by the manufacturer. β-galactosidase activities were quantified as relative light units per second (RLU/s) using the Orion Microplate Luminometer.

### Virus spread in PBMCs

To assess the ability of Nef to promote viral spread, 2 × 10^5 ^pre-stimulated PBMC per well were sown out in 48-well dishes and infected with 293T cell derived virus stocks containing one 1 ng of p24 antigen. Aliquots of the cells were obtained at 3, 5 and 7 days post-infection and the number of virally infected GFP^+ ^cells was determined by flow cytometric analysis.

### Phylogenetic analysis

**Figure 1:**
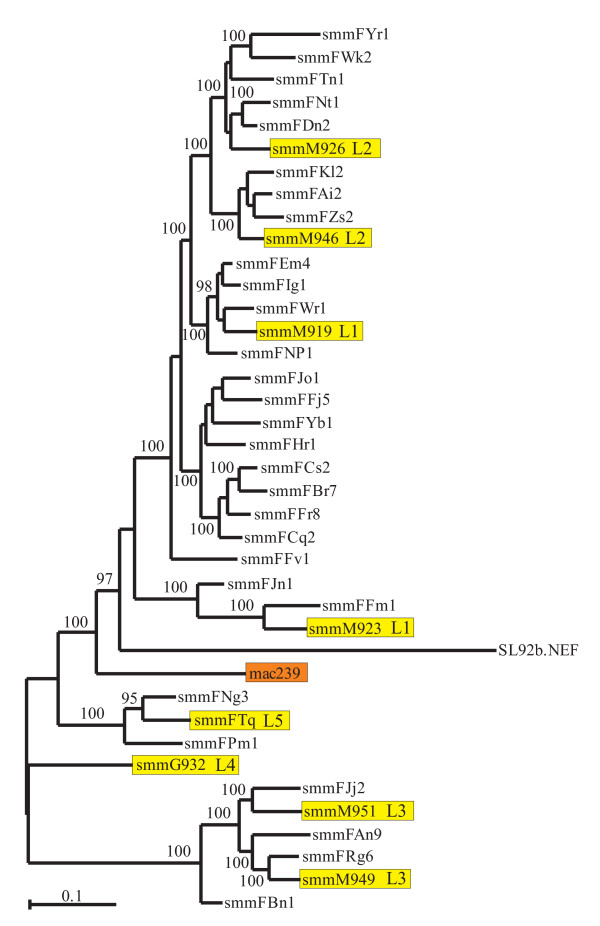
**Evolutionary relationships among HIV-2 and SIVsmm Nef sequences**. SIVsmm *nef *alleles newly analyzed in the present study and the SIVmac239 *nef *are highlighted by yellow and orange boxes, respectively. The tree in was inferred by the Bayesian method [48] MrBayes [49] using 10 million generations and the HKY model [50] with gamma distributed rates at sites [51] and burn in of 25%. Numbers on branches are percentage estimated posterior probabilities. Only those 95% and above are shown.

The *nef *sequences derived from the different lineages of SIVsmm analyzed in the present were compared to published SIVsmm *nef *sequences (accession numbers AF334679, DQ408682 to DQ408725 and EU636907 to EU636923). They were aligned using ClustalW [47]. The tree in Fig. 1 was inferred by the Bayesian method [48] in MrBayes [49] using 10 million generations and the HKY model [50] with gamma distributed rates at sites [51] and burn in of 25%. Average standard deviation of split frequencies (< 0.003) was checked to see that a sufficient number of generations had been run.

### Statistical methods

The activities of *nef *alleles were compared using a two-tailed Student's t test. The PRISM package version 4.0 (Abacus Concepts, Berkeley, CA) was used for all calculations.

### GenBank accession numbers

The GenBank accession numbers for the SIVsmm nef sequences are FJ943640 to FJ943647.

## Results

### Generation of viral constructs coexpressing Nefs from different SIVsmm lineages and eGFP

Extensive studies of SIVsmm diversity identified no less than nine different phylogenetic lineages of this virus in the animal cohorts housed at the Yerkes and Tulane National Primate Research Centers [13,21]. To assess whether SIVsmm shows lineage-specific differences in Nef function, we cloned *nef *alleles from 8 animals known to belong to five different clades based on the analysis of their *gag*, *pol *and *env *sequences [13,21] in bulk into an HIV-1 NL4-3-based IRES-eGFP proviral vector co-expressing Nef and eGFP from a bi-cistronic RNA. To generate these proviral constructs, the 3' end of the HIV-1 *env *gene and the SIVsmm *nef *alleles were fused by splice-overlap-extension (SOE) PCR using outer primers containing unique *HpaI *and *MluI *restriction sites and overlapping inner primers and cloned in bulk into the proviral constructs. As summarized in Table 1, the *nef *alleles represented SIVsmm lineages 1 (M919, M923), 2 (M926, M946), 3 (M949, M951) and 4 (G932). *Nef *genes from an L5 SIVsmm strain (FTq) were amplified from the supernatant of an infected SM PBMC culture. We found that sequences obtained by direct analysis of the PCR products and bulk inserts in the NL4-3-based IRES-eGFP vector were indistinguishable (data not shown). As further control, we sequenced three individuals' proviral clones for each of the eight animal samples. We found that all 24 proviral constructs encoded intact *nef *open reading frames and that these sequences were closely related to those obtained by direct sequencing of the corresponding PCR fragments and formed animal-specific clades for each of the eight SIVsmm strains. These results verified the accuracy of the proviral constructs and showed that the frequency of defective *nef *alleles is low.

### SIVsmm nef sequence and phylogenetic analysis

**Figure 2:**
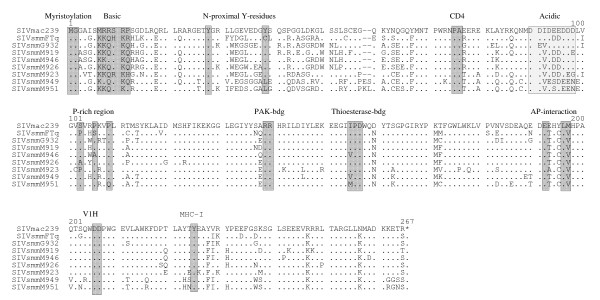
**Alignment of SIV and HIV-2 Nef sequences**. The SIVmac239 sequence is shown in the upper panel for comparison. Some conserved sequence elements in Nef, including the N-terminal myristoylation signal, N-proximal tyrosines (52–54), PA residues known to be critical for CD4 down-modulation by 239 wt Nef (58), the acidic and proline-rich regions, a diarginine motif, a C-proximal adaptor-protein (AP) interaction site (52), a diacidic putative VlH binding site and a Y residue involved in MHC-I down-regulation (52, 56,57) are indicated schematically. Dots indicate identity with the 239 wt Nef sequence and dashes gaps introduced to optimize the alignment.

Phylogenetic analyses showed that the proviral NL4-3 IRES/eGFP plasmid preparations contained highly divergent *nef *alleles that belonged to distinct clusters (Fig. 1). Importantly, this analysis demonstrated that all sequences were animal specific, thus confirming the authenticity of the amplified *nef *genes and excluding cross-sample PCR contaminations. Closer examination of the amplified sequences showed that all *nef *genes predicted full-length proteins of 265 or 267 amino acid residues (Fig. 2). The N- and C-termini of the SIVsmm Nef amino acid sequences were highly variable, whereas the central core region was well conserved. Some domains and protein interaction sites known to be relevant for HIV-1 function [52], such as the N-terminal myristoylation signal, an N-proximal basic region involved in membrane targeting, an acidic region, a di-arginine motif, a "di-leucine-based" (EXXXLM/V) adaptor-protein interaction site in the C-proximal flexible loop and a di-acidic putative V1H binding site were preserved in all SIVsmm Nef sequences (Fig. 2). In contrast, N-proximal Y residues, proposed to represent endocytosis signals [53,54], and the "proline-rich" region, which is highly conserved and involved in the interaction with cellular kinases in HIV-1 Nefs [55], differed in several SIVsmm Nef sequences. In contrast, Y223, which is critical for MHC-I down-modulation by SIVmac Nef [56,57] and residues P73A74 and D204, critical for CD4 down-modulation and efficient replication of SIVmac239 [58], were generally conserved at the corresponding position. Altogether, the examination of the *nef *sequences from the different clades of SIVsmm did not reveal any peculiar abnormalities.

### Nef alleles from different clades of SIVsmm modulate human receptors

**Figure 3:**
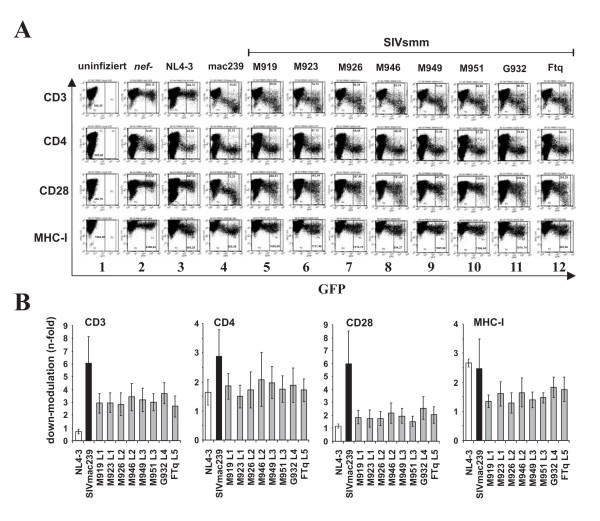
**Modulation of receptor expressed by human PBMC by SIVsmm *nef *alleles**. (A) PBMCs (panels A-D) were transduced with HIV-1 NL4-3 constructs coexpressing the indicated *nef *alleles and GFP and assayed for surface expression of CD4, CD3, CD28 and MHC-I. The ranges of eGFP expression used to calculate receptor modulation are indicated. (B) Quantitative assessment of SIVsmm Nef-mediated down-modulation of the indicated cellular receptors. The animal specifications and SIVsmm lineages are indicated and the activities obtained using the SIVmac Nef are shown for comparison. Values give averages ± SD derived from three independent experiments. The asterisks denote a statistical significance (P < 0.05) between SIVmac239 and SIVsmm Nef activity. All SIV *nef *alleles were significantly (P < 0.001) more active than that of HIV-1 in modulating CD3, whereas the HIV-1 *nef *was more active (P < 0.05) than all SIVsmm *nefs *in down-regulation of MHC-I.

**Figure 4:**
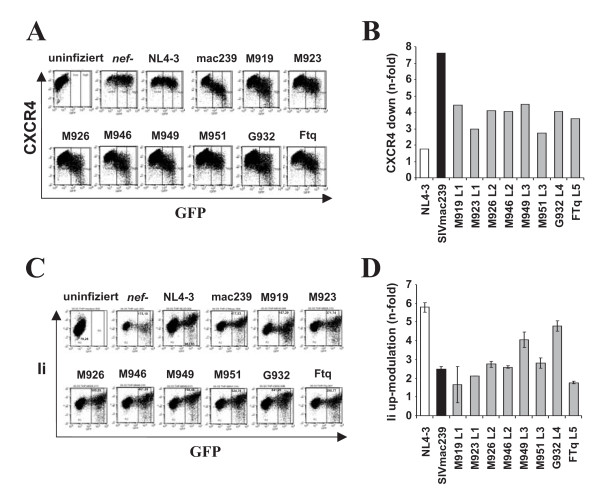
**Modulation of CXCR4 and the MHC-II associated invariant chain**. (A) Jurkat T cells were transduced with HIV-1 NL4-3 Nef/eGFP constructs and assayed for surface expression of CXCR4. The range of eGFP expression used to calculate CXCR4 modulation is indicated. (B) Quantitative assessment of down-modulation of CXCR4 by the indicated Nef proteins. (C) THP-1 cells were transduced with HIV-1 NL4-3 constructs coexpressing the indicated *nef *alleles and GFP and assayed for surface expression of Ii. The ranges of eGFP expression used to calculate receptor modulation are indicated. (D) Quantitative assessment of SIVsmm Nef-mediated down-modulation of Ii. Values give averages ± SD derived from five independent experiments. The asterisks denote a statistical significance (P < 0.05) between HIV-1 and SIV Nef activity.

To compare the potency of *nef *alleles from different SIVsmm lineages in down-modulating CD3, CD4, CD28 and MHC-I, and in up-regulating Ii, we transduced human PBMC with the proviral constructs and analyzed them by flow cytometry (Fig. 3A). As reported previously [30], the proviral NL4-3 Nef/eGFP constructs have the advantage that infected cells co-express Nef and eGFP from single bicistronic RNAs, thus allowing to correlate Nef and receptor expression levels (Fig. 3A). To ensure that the *nef *alleles were representative for each of the eight infected SMs investigated, they were cloned in bulk into the proviral vector and viral stocks were derived from ≥50 independent transformants (data not shown). For comparison, we also analyzed clones expressing an individual primary SIVsmm *nef *allele from each animal. The results confirmed the data obtained from the analyses of Nef function in bulk (data not shown). As expected, the control HIV-1 NL4-3 construct containing a disrupted *nef *gene only affected CD4, since Vpu and Env also reduce its expression at the surface of infected T cells (Fig. 3, column 2). In agreement with published data [30], the NL4-3 Nef did not down-modulate TCR-CD3 (Fig. 3A, column 3). In contrast, the 239 wt and the eight SIVsmm Nefs modulated the surface expression of all four receptors investigated (Fig. 3A, columns 4–12). Quantitative analyses demonstrated that Nefs from all five lineages of SIVsmm showed similar potency in down-modulating CD3, CD4, MHC-I and CD28 (Fig. 3B). Notably, the 239 wt Nef was more active than all SIVsmm Nefs in receptor modulation. This difference was particularly striking in Nef functions involved in T cell receptor (TCR) signalling. SIVsmm Nefs generally reduced CD3 cell surface expression about 3-fold, whereas the 239 wt Nef caused 6-fold down-modulation. The functional difference in down-modulation of the co-stimulatory factor CD28 was even more pronounced: all SIVsmm Nef alleles caused only about 2-fold lower levels of CD28 surface expression, whereas the 239 wt Nef reduced it by 6-fold (Fig. 3B). We also examined the effect of the SIVsmm *nef *alleles on CXCR4, which is down-modulated by many SIV and (to a lesser extend) HIV-1 Nefs to inhibit T cell migration [59]. This analysis was performed in Jurkat cells (Fig. 4A) instead of PBMCs because the latter express very low levels of CXCR4 upon PHA stimulation precluding the meaningful analysis of the Nef function (data not shown). We found that the 239 wt Nef down-modulated CXCR4 by 7.6-fold, whereas all SIVsmm Nefs had only 3.0- to 4.5-fold effects (Fig. 4B). Thus, *nef *alleles from all five lineages of SIVsmm modulate human receptors involved in TCR signalling and T cell migration, albeit generally with lower efficiency than the 239 wt Nef. Unfortunately, no SIVsmm-specific Nef antibodies are available. Thus, the Nef expression levels could not be determined, although the fact that all *nef *alleles were functionally active implies that they are efficiently expressed.

It has been previously shown that HIV and SIV Nefs up-regulate Ii, most likely to impair MHC-II antigen presentation [29,60]. We transduced the human monocytic leukemia THP-1 cell line with the NL4-3 Nef-IRES/eGFP constructs to study Ii up-modulation because it shares many properties with human monocyte-derived macrophages [61] and expresses high levels of MHC-I and MHC-II. As expected [60], THP-1 cells infected with the HIV-1 construct expressing the NL4-3 *nef *allele showed strongly enhanced levels of Ii surface expression (Fig. 4C). The SIVsmm Nefs varied substantially in their ability to up-modulate Ii. Those from M949 (L3) and G932 (L4) up-regulated Ii cell surface expression about 4-fold, whereas the M919 (L1) and FTq (L5) *nef *alleles caused less than 2-fold effects (Fig. 4D). Notably, the HIV-1 NL4-3 Nef was about 2-fold more effective than all SIV Nefs in up-modulating Ii. Taken together, these data demonstrate that the ability of Nef to modulate various human receptors involved in TCR signalling and MHC antigen presentation is conserved between the different lineages of SIVsmm. Our results also strongly suggest that the SIVmac239 Nef became more effective in some of these functions during its adaptation to rhesus macaques.

### SIVsmm Nefs suppress T cell activation

**Figure 5:**
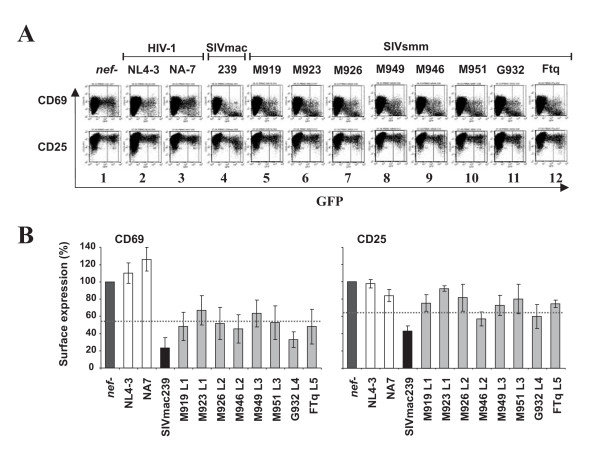
**Modulation of T activation by SIVsmm *nef *alleles**. **(A) **PBMCs were transduced with replication-competent VSV G pseudotyped HIV-1 particles expressing either GFP alone (panel 1) or together with the HIV-1 NL4-3 and NA7 (panels 2 and 3), 239 wt (panel 4) or SIVsmm *nef *alleles (panels 5 to 12) and stained for CD69 and CD25 expression. Similar results were obtained in two independent experiments. (B) CD69 and CD25 expression levels on PBMCs infected with HIV-1 constructs coexpressing eGFP and the indicated *nef *alleles are shown relative to those measured on T cells transduced with the *nef *defective control virus (100%). Values give averages ± SD derived from three independent experiments. The asterisks denote a statistical significance (P < 0.05) between HIV-1 and SIV Nef activity.

We next examined the effect of the various SIVsmm *nef *alleles on the responsiveness of virally infected T cells to activation. It has been previously shown that Nefs that down-modulate TCR-CD3 to suppress the responsiveness of infected T cells to stimulation, whereas those that do not perform this function have little inhibitory effect or even render the cells hyper-responsive to stimulation [30,43,45,62,63]. In agreement with these previous results, the about 5-fold increase in the expression levels of the early T cell activation marker CD69 upon PHA stimulation was blocked in PBMC infected with the HIV-1 construct expressing the 239 wt Nef but not in those expressing the HIV-1 NL4-3 or NA7 Nef proteins (Fig. 5A, panels 2–4). Consistent with their lower activity in CD3 down-modulation, the SIVsmm Nefs suppressed the induction of CD69 expression less efficiently than the 239 wt Nef (Fig. 5B, left). Nonetheless, they interfered with early T cell activation substantially more severely than those of HIV-1. In comparison, the SIVsmm Nefs had only weak inhibitory effects on the induction of CD25 at 2 days post-stimulation, whereas PBMC expressing the 239 wt Nef showed markedly (about 2.5-fold) reduced surface levels of this late T cell activation marker (Fig. 5B, right).

**Figure 6:**
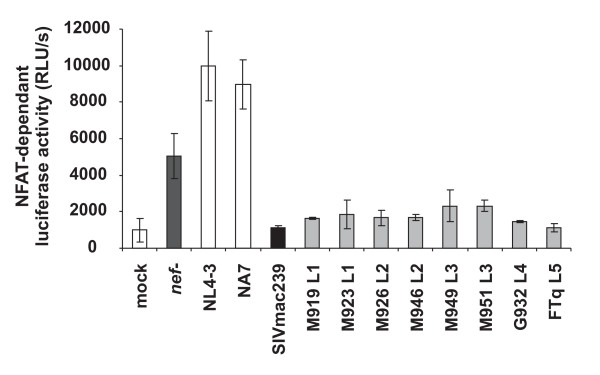
**SIVsmm *nef *alleles inhibit NFAT induction**. Analysis of Jurkat cells stably transfected with an NFAT-dependent reporter gene following transduction with the indicated HIV-1 Nef/eGFP constructs and subsequent stimulation with PHA. Levels of NFAT-dependent luciferase reporter activity are the average (± SD) of triple infections. The asterisks denote a statistical significance (P < 0.01) between HIV-1 and SIV Nef activity. Similar results were obtained in two independent experiments.

Activation of the nuclear factor of activated T cells (NFAT) plays a key role in T cell activation and regulates the transcription of many cellular genes, including that encoding for IL-2. It has been established that primate lentiviral Nef proteins differ fundamentally in their effect on the induction of NFAT [30,45]. To assess the effect of *nef *alleles from different lineages of SlVsmm on NFAT activation in virally infected cells, we transduced Jurkat T cells stably transfected with the luciferase reporter gene under the control of an NFAT-dependent promoter [45], with the proviral HIV-1 eGFP/Nef constructs and examined their responsiveness to activation. T cells infected with *nef *defective HIV-1 construct showed about 5-fold enhanced levels of NFAT activity upon PHA stimulation compared to mock infected cells (Fig. 6). As expected from previous studies [30,45], this increase was further enhanced by expression of the HIV-1 NL4-3 and NA7 Nefs but entirely blocked by the 239 wt Nef. Despite their reduced potency in CD3 and CD28 down-modulation, all SIVsmm Nefs suppressed the induction of NFAT-dependent luciferase activity almost as efficiently as the 239 wt Nef (Fig. 6). Compared to T cells infected with viral constructs containing HIV-1 *nef *alleles they expressed about 5- to 10-fold lower levels of NFAT-dependent luciferase activity. Thus, *nef *alleles from all five lineages of SIVsmm suppress the responsiveness of infected T cells to stimulation, albeit with lower potency than that of SIVmac239.

### SIVsmm Nefs enhance viral infectivity and replication

**Figure 7:**
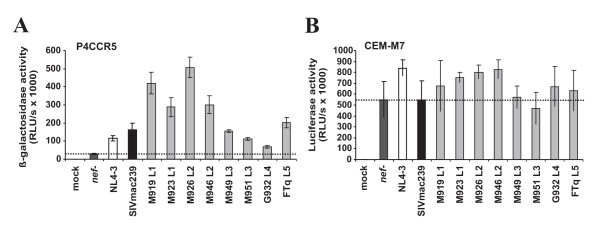
**SIVsmm *nef *alleles enhance viral infectivity**. Infectivity of HIV-1 NL4-3 variants containing the indicated *nef *alleles. (A) P4-CCR5 or (B) TZM-bl reporter cells were infected with HIV-1 NL4-3 IRES-eGFP constructs containing the indicated HIV and SIVsmm *nef *genes or a disrupted *nef *allele. Infections were performed in triplicate with two different virus stocks containing 1 ng p24 antigen. RLU/s, relative light units per second. All *nef *alleles (except G932) significantly (P < 0.05) enhanced virion infectivity in P4-CCR5 but not in CEM-M7 cells.

**Figure 8:**
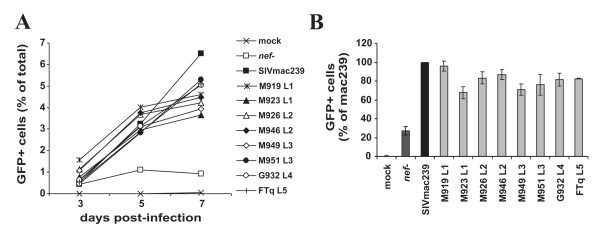
**Enhancement of viral replication by SIVsmm *nef *alleles**. (A) Replication kinetics of recombinant NL4-3 variants containing the indicated *nef *alleles in PBMCs are shown. Infections were performed using virus stocks containing 1 ng p24 antigen. The numbers of virally infected GFP^+ ^cells was measured by flow cytometric analysis. Similar results were obtained in two independent experiments (B) Cumulative numbers of HIV-1-infected GFP^+ ^cells detected in PBMC cultures at days 3, 5 and 7 post-infection. Shown are averages ± SD derived from three independent infections. All *nef *alleles significantly (P < 0.05) enhanced the number of HIV-1-infected cells compared to the *nef*-defective control virus.

The proviral HIV-1 IRES/eGFP constructs are replication-competent and hence allow to measure the ability of Nef to promote viral infectivity and replication. To assess the ability of the SIVsmm Nefs to enhance virion infectivity we infected P4-CCR5 [64], TZM-bl [65,66] and CEMx174 5.25 M7 (CEMx-M7) indicator cells with virus stocks containing 1 ng of p24 antigen derived from 293T cells transiently transfected with the different proviral constructs. We found that the control NL4-3 and 239 wt Nef proteins enhanced virion infectivity 4.0- and 5.6-fold, respectively, in P4-CCR5 cells (Fig. 7A) but had little if any enhancing effects in TZM-bl and CEM-M7 cells (Fig. 7B and data not shown). All eight SIVsmm *nef *alleles analyzed also enhanced viral infectivity in P4-CCR5 cells (Fig. 7A). However, although they showed similar potency in down-modulating CD3, CD4, CD28 and MHC-I (Fig. 3) and in suppressing T cell activation (Figs. 5 and 6) they varied substantially in their ability to promote virion infectivity (Fig. 7). The L1 and L2 M919, M923, M926 and M946 Nefs were more effective than the NL4-3 and 239 wt Nefs and increased virus infection between 10.0- and 17.4-fold (Fig. 7A). In comparison, the L4 G932 Nef was poorly active in enhancing virion infectivity (2.4-fold enhancement) and the remaining L3 and L5 Nefs showed an intermediate phenotype. Next, we investigated whether the eight SIVsmm bulk *nef *alleles promote viral spread in human PBMC cultures. We found that all SIVsmm Nefs enhanced viral replication with 239wt-like efficiency (Fig. 8A). In contrast to their differential effects on virion infectivity in P4-CCR5 cells, the eight SIVsmm *nef *alleles did not differ significantly in their ability to promote viral spread in human PBMC (Fig. 8B). This is consistent with previous results showing that the efficiency of HIV-1 replication does not correlate with the ability of Nef to enhance the infectivity of progeny virions [67,68]. Thus, *nef *alleles from all five lineages of SIVsmm are capable to promote viral spread and infectivity in human cells without adaptive changes.

### Subgroup-specific analysis of previously investigated SIVsmm Nefs

**Figure 9:**
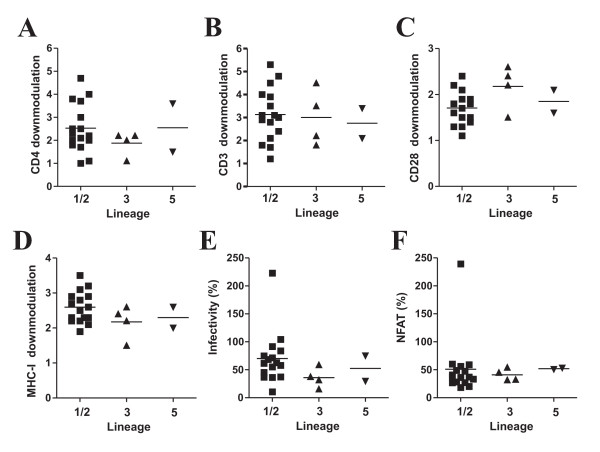
**SIVsmm *nef *alleles derived from SMs with high or low CD4^+ ^T cell counts do not show significant lineage-specific differences in Nef function**. (A-D) Modulation of cellular receptors by primary *nef *alleles derived from different lineages of SIVsmm. Each symbol represents n-fold down-modulation of the indicated receptor molecule by one of the 22 NL4-3 recombinants expressing primary bulk SIVsmm *nef *alleles analyzed. Similar results were obtained in two independent experiments. L1 and L2 nef alleles were combined since they did not form two distinct clusters (Fig. 1). (E) Enhancement of virion infectivity by SIVsmm *nef *alleles. P4-CCR5 indicator cells were infected with HIV-1 NL4-3 IRES-eGFP constructs containing the indicated SIVsmm *nef *genes or a defective *nef *allele. Infections were performed in triplicate with two different virus stocks containing 1 ng p24 antigen. Each symbol represents the average value of the 6 measurements compared to the infectivity of the virus expressing the SIVmac239 Nef (100%). (F) Levels of NFAT-dependent luciferase reporter activity in Jurkat T cell cultures infected with HIV-1 variants expressing *nef *alleles from SIVsmm-infected SMs compared to the *nef *defective control. The luciferase reporter activities represent the average (± SD) of triple infections. Similar results were obtained in two independent experiments. For clarity animal FYb was excluded from the analysis in the right panel.

The data described above show that Nefs from different lineages of SIVsmm are basically capable to modulate various receptors and to enhance viral infectivity and replication in human-derived cells. As a first step to elucidate whether different lineages of SIVsmm show quantitative differences in Nef function, we re-analyzed data derived from the functional analysis of 22 *nef *alleles from 14 naturally infected SMs with low (<500/μl) and eight animals with high (>500/μ1) CD4^+ ^T cell counts [42]. This study showed that inefficient down-modulation of CD3 and MHC-I by Nef is associated with low CD4^+ ^T cell counts in this natural simian host of SIV but did not assess whether these SIVsmm Nefs show lineage-specific differences in Nef function. Phylogenetic analyses showed that two of the 22 *nef *sequences clustered with the L5 FTq sequence and four with the L3 M951 and M949 sequences (Fig. 1). These L3 and L5 *nef *sequences formed two distinct clusters. In comparison, the remaining 16 SIVsmm *nef *sequences were more closely related to those derived from the more common SIVsmm L1 and L2 strains (Fig. 1). These *nef *sequences showed a high degree of genetic diversity and L2 *nef *alleles fell within the L1 cluster. Therefore, we grouped them together and refer to them herein after as L1/2 Nefs. Examination of the functional data showed that, on average, L1/2, L3 and L5 SIVsmm Nefs showed similar activities in down-modulating CD4 and CD3 (Fig. 9A, B). All 22 SIVsmm *nef *alleles had only weak effects on CD28, although those derived from L3 were usually a little more active than others (Fig. 9C). These lineages of SIVsmm did also not show a significant differences in Nef-mediated modulation of MHC-I (Fig. 9D). In agreement with the results shown in Fig. 8, L1/2 *nef *alleles were, on average, more potent in enhancing virion infectivity than L3 Nefs (Fig. 9E). As reported previously [42], Nef alleles from a single SIVsmm-infected SM with very low numbers of CD4^+ ^T cells rendered T cells hyper-responsive to activation, similarly to those of HIV-1 (Fig. 9F). The remaining SIVsmm Nefs all suppressed NFAT induction and did not show significant lineage-specific differences in this activity (Fig. 9F). Altogether, these results demonstrate that SIVsmm *nef *alleles from SMs with different numbers of CD4^+ ^helper T cells show substantially functional differences but these are strain rather than lineage dependent.

## Discussion

In the present study, we show that *nef *alleles from different lineages of SIVsmm modulate the surface expression of human CD4, CD3, CD28, MHC-I and Ii molecules, suppress T cell activation and enhance viral spread and infectivity. This result is in agreement with our previous findings showing that SIVsmm and SIVcpz *nef *alleles [43,69] but also those derived from SIVs that have not been found in humans [30] do not require adaptive changes to be functionally active in human cells. We did not find any significant lineage-specific differences in SIVsmm Nef function. Thus, although a larger number of *nef *alleles from all clades of SIVsmm must be analyzed to definitely exclude this possibility, our results suggest that lineage-specific differences in SIVsmm Nef function do not play a major role in the virological and immunological features of natural SIVsmm infection in SMs. In comparison, strain-dependent differences in Nefs ability to facilitate viral immune evasion and to promote viral spread may significantly affect the fitness and pathogenicity of SIVsmm in its natural SM, human and experimental macaque hosts.

One remarkable observation was that SIVsmm *nef *alleles were generally less potent in down-modulating various human receptors and in suppressing T cell activation than that of SIVmac239. This difference was not due to the fact that the bulk *nef *preparations contained a high frequency of inactivating point mutations, since all 24 individual proviral constructs encoded intact *nef *open reading frames and because the activity of the individual *nef *alleles recapitulated that of the bulks. Altogether, SIVsmm *nef *alleles from about 40 different SMs and belonging to five different lineages have been functionally analyzed to date [42,43]. The fact that SIVsmm Nefs are generally less active in modulating CD3, CD28 and CXCR4 than that of SIVmac239 suggests that the latter evolved to become more active in suppressing the migration and activation of infected T cells during its adaptation to rhesus macaques. At first view this may seem counterintuitive since SIVmac239 infection of rhesus macaques is associated with high levels of chronic T cell activation and rapid loss of CD4^+ ^T cell loss and progression to simian AIDS [35]. It is conceivable, however, that for effective viral persistence in their respective hosts, HIV and SIV must balance the activation of virally infected T cells to levels that are high enough to ensure efficient proviral transcription but also so low that they do not cause apoptosis before the viral replication cycle is completed. The necessity to carefully adjust the responsiveness of virally infected T cells to activation to achieve this balance most likely explains why primary SIVsmm Nefs show only moderate activity in functions affecting the interaction and stimulation of T cells by APCs. The experimental macaque host reacts with much higher levels of immune activation and T cell activation to SIV infection than the natural SM host [70]. It is thus plausible that SIVsmm/SIVmac may have evolved not only to persist efficiently at high levels but also to become more active in suppressing T cell activation in macaques to compensate for the aggravated immune response in this new host.

One limitation of the present study is that the data on SIVsmm Nef function were derived using an HIV-1-based proviral construct in human derived cells. We have previously shown, however, that HIV-1, SIVmac and SIVagm derived proviral constructs expressing various HIV and SIV *nef *alleles exhibited the same phenotype in human and SM PBMC [30]. Thus, the effect of Nef on receptor modulation and T cell activation is independent of the proviral context and conserved in target cells from divergent primate species. Moreover, one goal of the present study was to assess whether lineage-dependent differences in Nef function in human cells may have affected the fitness and hence the subsequent spread of SIVsmm/HIV-2 in the new human host. Lack of Nef function could potentially play a relevant role because Nef is required for effective viral persistence [32-35] and the efficiency of sexual viral transmission correlates with the viral load. Our finding that *nef *alleles from all five lineages of SIVsmm analyzed modulated various human receptors, suppressed T cell activation and promoted viral infectivity in human-derived cells suggests that this was most likely not the case. However, larger numbers of *nef *alleles from the different lineages of SIVsmm need to functionally to exclude the possibility that some (perhaps subtle) functional differences do exist. For example, L1 and L2 *nef *alleles usually enhanced virion infectivity more efficiently than those derived from L3. Whether or not it is just coincidence that SIVsmm L1 and L2 are more widespread and associated with higher viral loads compared to L3 in the animal cohorts housed at the YNPRC and TNPRC [21] remains to be determined. Previous results in the SIVmac/macaque model suggest that Nef-mediated enhancement of virion infectivity contributes to efficient viral replication *in vivo *[58,70], although the fact that the *nef *alleles used in these studies also differed in other functional aspects precludes definitive conclusions.

Another issue that warrants further study is the previous finding that 3 out of 4 SMs infected with L5 SIVsmm strains showed a significant loss of CD4^+ ^T cells, whereas this only observed in about 10–15% of animals infected with SIVsmm lineages 1, 2 and 3 [21,22]. Since inefficient Nef-mediated downmodulation of CD3 and MHC-I correlates with numbers of CD4^+ ^T cells in natural SIVsmm infection [42] it was tempting to speculate that L5 Nefs may be poorly active in these functions. In the present study we did not observe a particularly low activity of L5 Nef in modulating CD3 or MHC-I. However, only three L5 *nef *alleles were available for functional analyses. Furthermore, the FTq *nef *that has not been analyzed in the previous study [42] was derived from the only SIVsmm L5 infected SM with normal CD4^+ ^T cell counts (~850/μl) and no plasma sample was available from the animals with the lowest number of CD4^+ ^T cells. Finally, the comparison of the functional activity of the remaining two L5 Nefs with those derived from other lineages shown in Fig. 9 is not suitable to address the question because SIVsmm L1, L2 and L3 infected SMs with low CD4^+ ^T cell counts are strongly over-represented in this set of *nef *alleles [42]. The fact, that these previously examined *nef *alleles were derived from SMs selected based on their different CD4^+ ^T cell counts may also explain why they are functionally more divergent than the newly analyzed *nef *alleles.

The fact that primary *nef *alleles from all lineages of SIVsmm analyzed are functional in human-derived cells suggests that Nef facilitated HIV-2 to maintain high viral loads and spread in the new human host without requiring adaptive changes. In contrast to natural SIVsmm infection, HIV-2 is associated with AIDS in a significant number of infected individuals, although it causes lower levels of immune activation and is substantially less pathogenic than HIV-1 [71,72]. Furthermore, in strict contrast to natural SIV infections, non-progressing HIV-2 infections are typically associated with low viral loads [73,74] and hence most likely also with ineffective virus transmission. In fact, HIV-2 has spread substantially less efficiently in the human population than HIV-1 and recent findings suggest that the HIV-2 epidemic is now declining [75]. Currently, too little information is available to assess how SIVsmm/HIV-2 Nef function evolved in the new human host. Preliminary data suggest, however, that the prevalence of defective *nef *genes may be higher in HIV-2 than in SIVsmm and HIV-1 infections [42,43,76]. Furthermore, HIV-2 Nefs are usually less active in enhancing viral replication *in vitro *than both HIV-1 and SIVsmm Nefs [43] and HIV-2 isolates show lower replicative fitness compared to HIV-1 isolates in infected PBMC cultures [77]. To obtain further insights into the evolution of SIVsmm/HIV-2 Nef function after zoonotic transmission and its role in HIV-2 replication and pathogenesis it will be interesting to perform a systematic and comprehensive analysis of primary HIV-2 *nef *alleles from infected individual who are clinically well characterized. Taken together, our current knowledge shows that SIV and HIV *nef *alleles are usually functionally active in cells of a new host species, such as humans or macaques. However, the fine-tuning of Nef function to allow effective viral replication and spread without causing harm to the infected host is obviously difficult to achieve.

## Conclusion

Our analysis of *nef *alleles from different clades of SIVsmm shows that they are all capable to modulate the surface expression of various receptors and to enhance viral infectivity and replication in human derived cells. These results suggest that lack of these Nef functions was not the reason why only two of eight zoonotic transmission of SIVsmm from SMs to humans resulted in significant spread in the human population. Our finding that primary SIVsmm Nefs are generally only moderately active in functions that affect the migration of T cells and their responsiveness to stimulation most likely reflects the necessity for SIVsmm to curb T cell activation to levels that warrant effective viral replication without damaging the host immune system. Together with the results of previous studies [30,42,43,46,69], the present data show that SIV *nef *alleles from African non-human primate species are usually functional in human or macaque derived cells. Obviously, however, the fine-tuning of various Nef (and possibly other) functions to establish an elaborative well-balanced virus-host relationship similarly to that found in some natural SIV infections is also dependent on various host factors and seems difficult to achieve. Further studies with well characterized molecular SIVclones differing in the repertoire (e.g. the presence of *vpr*, *vpx*, *vpu *and *nef *gene) or function of their accessory genes in adapted and non-adapted monkey hosts are needed to achieve a better understanding of these complex virus-host interactions.

## Competing interests

The authors declare that they have no competing interests.

## Authors' contributions

Conceived and designed the experiments: GS BHH CA FK. Performed the experiments: JS HL MS. Analyzed the data: JS HL CA FK. Contributed reagents/materials/analysis tools: GS BHH CA FK. Wrote the paper: FK.
